# First Evidence of the Protective Effects of 2-Pentadecyl-2-Oxazoline (PEA-OXA) in In Vitro Models of Acute Lung Injury

**DOI:** 10.3390/biom13010033

**Published:** 2022-12-24

**Authors:** Aniello Schiano Moriello, Fiorentina Roviezzo, Fabio Arturo Iannotti, Giuseppina Rea, Marco Allarà, Rosa Camerlingo, Roberta Verde, Vincenzo Di Marzo, Stefania Petrosino

**Affiliations:** 1 Endocannabinoid Research Group, Institute of Biomolecular Chemistry, National Research Council, 80078 Pozzuoli, Italy; 2 Epitech Group SpA, Saccolongo, 35100 Padova, Italy; 3 Department of Pharmacy, University of Naples Federico II, 80138 Naples, Italy; 4 Microenvironment Molecular Targets, National Cancer Institute G. Pascale Foundation, IRCCS, 80131 Naples, Italy; 5 Cellular Biology and Biotherapy-Research Department, National Cancer Institute G. Pascale Foundation, IRCCS, 80131 Naples, Italy; 6 Canada Excellence Research Chair on the Microbiome-Endocannabinoidome Axis in Metabolic Health, CRIUCPQ and INAF, Faculties of Medicine and Agriculture and Food Sciences, Université Laval, Quebec City, QC G1V 4G5, Canada

**Keywords:** acute respiratory distress syndrome, anti-inflammatory, endocannabinoids, fibrosis, lung epithelial cells, palmitoylethanolamide, 2-pentadecyl-2-oxazoline

## Abstract

Acute respiratory distress syndrome (ARDS) is a serious inflammatory lung disorder and a complication of SARS-CoV-2 infection. In patients with severe SARS-CoV-2 infection, the transition to ARDS is principally due to the occurrence of a cytokine storm and an exacerbated inflammatory response. The effectiveness of ultra-micronized palmitoylethanolamide (PEA-um) during the earliest stage of COVID-19 has already been suggested. In this study, we evaluated its protective effects as well as the effectiveness of its congener, 2-pentadecyl-2-oxazoline (PEA-OXA), using in vitro models of acute lung injury. In detail, human lung epithelial cells (A549) activated by polyinosinic–polycytidylic acid (poly-(I:C)) or Transforming Growth Factor-beta (TGF-β) were treated with PEA-OXA or PEA. The release of IL-6 and the appearance of Epithelial–Mesenchymal Transition (EMT) were measured by ELISA and immunofluorescence assays, respectively. A possible mechanism of action for PEA-OXA and PEA was also investigated. Our results showed that both PEA-OXA and PEA were able to counteract poly-(I:C)-induced IL-6 release, as well as to revert TGF-β-induced EMT. In addition, PEA was able to produce an “entourage” effect on the levels of the two endocannabinoids AEA and 2-AG, while PEA-OXA only increased PEA endogenous levels, in poly-(I:C)-stimulated A549 cells. These results evidence for the first time the superiority of PEA-OXA over PEA in exerting protective effects and point to PEA-OXA as a new promising candidate in the management of acute lung injury.

## 1. Introduction

Acute respiratory distress syndrome (ARDS) is the most devastating condition of acute lung injury, characterized by pulmonary edema, severe hypoxemia and impaired ability to eliminate CO_2_, and represents one among the most challenging clinical disorders of critical care medicine with high mortality [1,2,3]. A wide spectrum of risk factors associated with ARDS, such as pneumonia, bacterial or viral infection, transfusion of blood components, trauma, acute pancreatitis and drug reaction, can cause direct or indirect acute lung injury [2]. Among viral infections the current SARS-CoV-2 pandemic has arisen as a new risk factor in as much as it can affect the lower respiratory tract by causing ARDS [4]. In particular, in the early stage of COVID-19, the first cells infected by SARS-CoV-2 are the nasal ciliated cells [5]. Successively, if innate or adaptive responses are not able to clear the virus, the latter can spread from the nasal cavity to the lung via inhalation and thus infect alveolar cells [6,7], causing a diffuse alveolar damage that might progress to ARDS. Patients with severe COVID-19 exhibit systemic hyper-inflammation characterized by a cytokine storm, including an excessive release of pro-inflammatory mediators, such as interleukin (IL)-1, IL-6, IL-8 and tumor necrosis factor-alpha (TNF-α) [8], which in turn is responsible for ARDS. In addition, in the lungs of patients with severe COVID-19 the expansion of fibroblasts, which determines a degree of fibrosis that increases over the course of the disease [8], was found, causing or worsening lung injury and failure.

Palmitoylethanolamide (PEA), originally identified in egg yolk and subsequently in a wide variety of food sources, is highly recognized as an endogenous bioactive lipid, given its presence in most cells and tissues, from both animals and humans [9]. PEA is synthesized “on demand” in conditions of current or potential damage and is endowed with anti-inflammatory, analgesic and neuroprotective properties [10], which are mediated by several molecular and cellular mechanisms. One of these is the Autacoid Local Inflammation Antagonism (ALIA), through which PEA downregulates the degranulation of mast cells [11]. PEA is known to directly activate the peroxisome proliferator-activated receptor- (PPAR-α) [12] and the orphan G-protein-coupled receptor 55 (GPR55) [13] or to indirectly activate the cannabinoid receptors CB1 and CB2 [14,15] and the transient receptor potential vanilloid 1 (TRPV1) [16,17,18]. The indirect interaction of PEA with cannabinoid and vanilloid receptors is known as the “entourage effect”, since it is due to PEA increasing the levels of the endocannabinoids and endovanilloids, i.e., anandamide (AEA) and 2-arachidonoyl-glycerol (2-AG). The effect depends upon the inhibition or down-regulation of the AEA-hydrolyzing enzyme fatty acid amide hydrolase (FAAH) [14] or the stimulation of the activity of the biosynthesizing enzyme diacylglycerol lipase (DAGL) [15]. PEA tissue concentrations are altered during different neuro-inflammatory disorders, suggesting that (i) increased levels might represent a compensatory mechanism to restore homeostasis, while (ii) decreased levels might contribute to the etiology of the disease [9,10,19]. For these reasons, the exogenous application of PEA could be required to potentiate the endogenous protective mechanisms, when the endogenous production of PEA is insufficient o lacking [20,21].

Recently, a natural congener of PEA, 2-pentadecyl-2-oxazoline (PEA-OXA), identified in both green and roasted coffee beans [22], has been reported to have anti-inflammatory and anti-nociceptive properties in an experimental model of acute inflammatory pain [23], to reduce neuroinflammation in an experimental model of Parkinson’s disease [24] and to exert neuroprotective effects in different neuroinflammatory conditions associated with spinal and brain trauma in mice [25]. It has been hypothesized that PEA-OXA could exert its protective role by inhibiting the enzyme responsible for PEA catabolism, *N*-acyl-ethanolamine-hydrolyzing acid amidase (NAAA), resulting in an increase in the endogenous levels of PEA [23].

Therefore, given the ability of PEA and its congener PEA-OXA to exert important anti-inflammatory and protective effects, the present work aimed to investigate their potential effectiveness in in vitro models of acute lung injury, reproducing the clinical conditions of SARS-CoV-2 infection. In particular, this study was based on two currently well-established facts, i.e., i) IL-6 is a key contributor of the cytokine storm observed in SARS-CoV-2 infection-associated hyperinflammation and multiorgan failure [8,26], suggesting that this cytokine is a promising marker and an efficacious therapeutic target for the treatment of COVID-19 [26,27]; and ii) ultramicronized PEA (PEA-um) is a promising adjuvant to be assumed in the earliest stage of COVID-19, since a reduction in the inflammatory state has been demonstrated both in cultured murine alveolar macrophages activated by the SARS-CoV-2 Spike Protein [28] and in a randomized clinical trial [29] carried out with this formulation of PEA. 

To achieve our objectives, here we used the human lung epithelial cell line A549 to reproduce: (i) a viral infection resulting in IL-6 cytokine release and (ii) Epithelial–Mesenchymal Transition (EMT) mechanisms resulting in lung fibrosis.

## 2. Materials and Methods

All reagents were purchased from Sigma-Aldrich (Milano, Italy) unless otherwise stated. Pentadecyl-2-oxazoline (PEA-OXA) and palmitoylethanolamide in an ultra-micronized formulation (referred to as PEA hereafter) were obtained from Epitech Group SpA (Saccolongo, Padova, Italy). Polyinosinic–polycytidylic acid (poly-(I:C)) was purchased from InvivoGen (Aurogene, Roma, Italy). 5′-Iodoresiniferatoxin (IRTX) and GW6471 were purchased from Tocris Bioscience (Space Import-Export, Milano, Italy). The deuterated standards—[^2^H]_8_-AEA, [^2^H]_5_-2-AG and [^2^H]_4_-PEA—were purchased from Cayman Chemical (Cabru, Arcore, Italy). The human lung epithelial cell line (A549) was purchased from LGC Standards (Milano, Italy). The human IL-6 ELISA Kit and transforming growth factor beta (TGF-β) were purchased from Abcam (Prodotti Gianni, Milano, Italy). Total mRNA was isolated from A549 cells using Trizol Reagent (Thermo Fisher, Milano, Italy) following the manufacturer’s instructions. cDNA preparation from RNA was performed using iScript Reverse Transcription enzyme (Biorad, Milano, Italy). Specific primer sequences were designed using Primer3 Software (https://primer3.ut.ee/, accessed on 28 November 2022) and synthetized by Eurofin (Milano, Italy).

### 2.1. Cell Culture

A549 cells were grown in Dulbecco’s Modified Eagle Medium (DMEM) complemented with penicillin (400 U mL^−1^), streptomycin (50 mg mL^−1^) and 10% Fetal Bovine Serum (FBS), in the presence of a 5% CO_2_ atmosphere at 37 °C, plated on 100 mm diameter Petri dishes.

### 2.2. Poly-(I:C)-Induced Inflammatory Response in A549 Cells

A549 cells were plated into 24-well culture dishes at a cell density of 2 × 10^5^ cells per well for 1 day at 37 °C in a 5% CO_2_ atmosphere. After 1 day, A549 cells were stimulated with poly-(I:C) (100 μg mL^−1^) or vehicle (water) and incubated for 6 h at 37 °C in a 5% CO_2_ atmosphere. Poly-(I:C)-stimulated A549 cells were treated with PEA-OXA (0.1, 1 and 10 µM), PEA (0.1, 1 and 10 μM) or vehicle (dimethyl sulfoxide or methanol, respectively) and incubated for the indicated time. Poly-(I:C)-stimulated A549 cells were also treated with a TRPV1 antagonist, IRTX (0.1 μM), or PPAR-α antagonist, GW6471 (1 µM), in the presence or absence of PEA-OXA (10 µM) or PEA (10 μM) and incubated for the indicated time. After 6 h, the supernatants were collected, and the amounts of produced IL-6 were measured by using a human IL-6 ELISA kit according to the manufacturer’s instructions (Abcam) and by using a reader Glomax^®^ Explorer (Promega, Milano, Italy). Data are expressed as picograms per milliliter of IL-6.

### 2.3. RNA Extraction and Quantitative PCR (qPCR)

Total RNA was isolated from A549 cells using TRIzol Reagent (cat# 15596018, Life Technologies, Milano, Italy) and reacted with DNase-I (cat# AMPD1, Merk, Milano, Italy) for 15 min at room temperature, followed by spectrophotometric quantification. Subsequently, the RNA integrity number (RIN) for each RNA sample was analyzed on an Agilent 2100 bioanalyzer (Roma, Italy). Purified RNA was reverse-transcribed by the use of the iScript cDNA Synthesis Kit (cat# 1708841, Bio-Rad, Milano, Italy). Quantitative PCR (qPCR) was carried out in a real-time PCR system CFX384 (Bio-Rad, Milano, Italy) using the SYBR Green PCR Kit (Cat# 1725274, Bio-Rad for mRNAs) detection technique and specific primer sequences (Table 1).

Quantitative PCR was performed on independent biological samples (*n* = 3). Each sample was amplified simultaneously in quadruplicate in a one-assay run with a nontemplate control blank for each primer pair to control for contamination or primer–dimer formation, and the cycle threshold (Ct) value for each experimental group was determined. The housekeeping gene ribosomal protein S16 was used to normalize the Ct values, using the 2^−ΔCt^ formula.

### 2.4. Quantification by Liquid Chromatography–Atmospheric Pressure Chemical Ionization–Mass Spectrometry (LC-APCI-MS) of the Endogenous AEA, 2-AG and PEA Levels in A549 Cells

A549 cells, plated in 6-well culture dishes at a cell density of 9 × 10^5^ cells per well, were stimulated with poly-(I:C) (100 μg mL^−1^) or vehicle (water) and treated in the presence or absence of PEA-OXA (10 µM), PEA (10 µM) or vehicle (dimethyl sulfoxide and methanol, respectively) and incubated for 6 h at 37 °C in a 5% CO_2_ atmosphere. After 6 h, cells and supernatants were collected and homogenized in a solution of chloroform/methanol/Tris-HCl 50 mM, pH 7.4 (2:1:1 by vol.) containing 10 pmol of [^2^H]_8_-AEA and 50 pmol of [^2^H]_5_-2-AG and [^2^H]_4_-PEA as internal standards. The lipid-containing organic phase was dried, weighed and pre-purified by open-bed chromatography on silica gel. Fractions derived by eluting the column with a solution of chloroform/methanol (90:10 by vol) were analyzed by LC-APCI-MS by using a Shimadzu (Shimadzu, Kyoto, Japan) High Performance Liquid Chromatography (HPLC) apparatus (LC-10ADVP) coupled with a Shimadzu (LCMS-2020) quadrupole MS via a Shimadzu APCI interface. LC-APCI-MS analyses of AEA, 2-AG and PEA were performed in the selected ion-monitoring (SIM) mode [30,31], using m/z values of 356 and 348 (molecular ion + 1 for deuterated and undeuterated AEA), 384.35 and 379.35 (molecular ion + 1 for deuterated and undeuterated 2-AG), and 304 and 300 (molecular ion + 1 for deuterated and undeuterated PEA). The AEA, 2-AG and PEA levels were determined on the basis of their area ratio with the internal standard signal areas to provide the amounts in pmol mg^–1^ of the lipid extract.

### 2.5. TGF-β-Induced Epithelial–Mesenchymal Transition in A549 Cells

A549 cells, harvested at 80% confluence and plated into 24-well culture dishes, were stimulated with TGF-β1 (2 ng mL^−1^) or vehicle (PBS) and incubated for 72 h at 37 °C in a 5% CO_2_ atmosphere. TGF-β-stimulated A549 cells were treated with PEA-OXA (10 µM), PEA (10 µM) or vehicle (dimethyl sulfoxide and methanol, respectively) and incubated for the indicated time. After 72 h, A549 cells were fixed with 70% ethanol/0.1% Triton for 30 min at 4 °C, treated with 5% BSA for 60 min at room temperature and then stained with primary antibodies, rabbit anti-human cytokeratin (clone ab9377, Abcam) and mouse anti-human vimentin V9 (clone ab8069, Abcam), overnight at 4 °C. The secondary antibodies, goat anti-rabbit Alexa fluor488 (Cell Signaling Technology Danvers, MA, USA) and goat anti-mouse Alexa fluor594 (Cell Signaling Technology Danvers, MA, USA), were incubated for 60 min at 4 °C, and DAPI (Sigma, Milano, Italy), used to stain the nucleus, was incubated for 7 min at room temperature. Appropriate isotype controls were used. The images were acquired with a fluorescence microscope (Zeiss, Milano, Italy) and AxionCam MRc5 (Zeiss, Milano, Italy).

### 2.6. Data Analysis

Each experiment was performed in at least 3–4 independent biological samples for each group. Data were expressed as means ± standard error of the mean (SEM). Statistical analyses were performed using GraphPad Prism software version 9.0 (GraphPad Software Inc., San Diego, CA, USA). One-way analysis of variance (ANOVA) followed by Tukey’s multiple comparison test was used for the analysis. *p* values < 0.05 were considered statistically significant. Figures were generated in GraphPad Prism software version 9.0.

## 3. Results

### 3.1. PEA-OXA and PEA Reduce Poly-(I:C)-Induced Release of Il-6 in Lung Epithelial Cells

**Figure 1:**
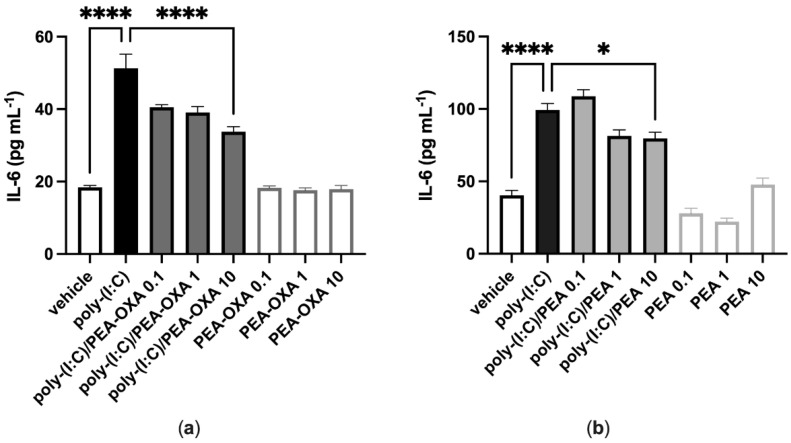
PEA-OXA and PEA reduce IL-6 release from poly-(I:C)-stimulated A549 cells. (**a**) IL-6 release was measured after stimulation of A549 cells with poly-(I:C) (100 μg mL^−1^) in the presence or absence of PEA-OXA (0.1, 1 and 10 μM) for 6 h at 37 °C in a 5% CO_2_ atmosphere; (**b**) IL-6 release was measured after stimulation of A549 cells with poly-(I:C) (100 μg mL^−1^) in the presence or absence of PEA (0.1, 1 and 10 μM) for 6 h at 37 °C in a 5% CO_2_ atmosphere. Each bar shows the mean ± SEM of independent experiments (*n* = 4). *p*-values were determined by ANOVA followed by Tukey’s multiple comparisons test. **** *p* < 0.0001 and * *p* < 0.05.

A549 cells stimulated with poly-(I:C) (100 μg mL^−1^ for 6 h) and treated with the vehicle of PEA-OXA or PEA significantly released IL-6, as compared to vehicle-stimulated A549 cells (Figure 1). PEA-OXA and PEA, at the highest concentration tested (10 μM), reduced the release of IL-6 from poly-(I:C)-stimulated A549 cells, as compared to poly-(I:C)-stimulated A549 cells treated with the vehicle of PEA-OXA or PEA (Figure 1). PEA-OXA (10 μM) was more effective (1.6-fold) than PEA (10 μM) in reducing the release of IL-6 from poly-(I:C)-stimulated A549 cells (53% and 33% of inhibition, respectively) (Figure 1). No effect on IL-6 release was observed when A549 cells were treated with PEA-OXA or PEA alone (0.1, 1 and 10 μM), i.e., in the absence of poly-(I:C), as compared to vehicle-treated A549 cells (Figure 1).

### 3.2. The Anti-Inflammatory Effect of PEA-OXA and PEA Is Not Reverted by Antagonism at the TRPV1 or PPAR-α Receptors in Lung Epithelial Cells

**Figure 2:**
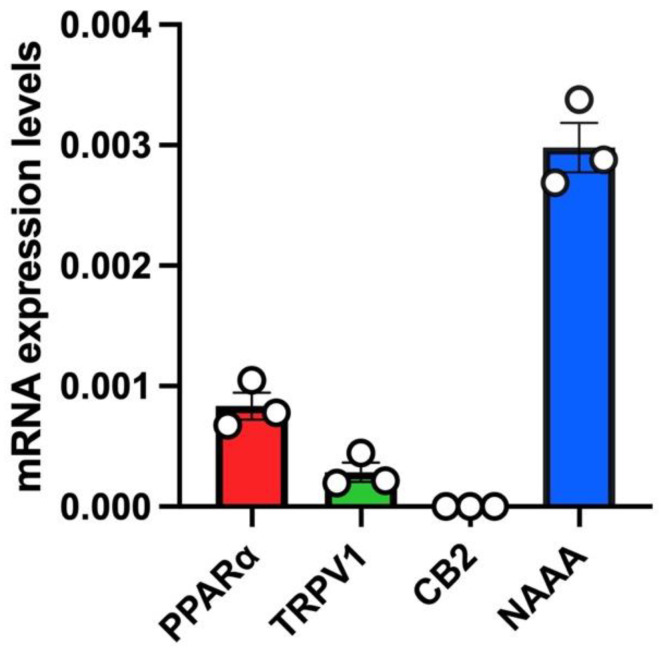
mRNA expression levels of PEA targets (PPARα, TRPV1, CB2) and PEA-catabolizing enzyme (NAAA) in A549 cells. Bar chart with individual points showing the mRNA expression levels of the indicated proteins (PPARα, TRPV1, CB2 and NAAA) measured in A549 cells. Each bar shows the mean ± SEM of 3 independent biological samples. Data are expressed using the 2^−Δct^ formula.

In untreated A549 cells, we a found robust mRNA expression of PPARα and a low relative mRNA expression of TRPV1 (Figure 2). Instead, no mRNA expression of CB2 was found in untreated A549 cells (Figure 2). Importantly, the highest mRNA expression levels in this cell line were found for NAAA (Figure 2).

**Figure 3:**
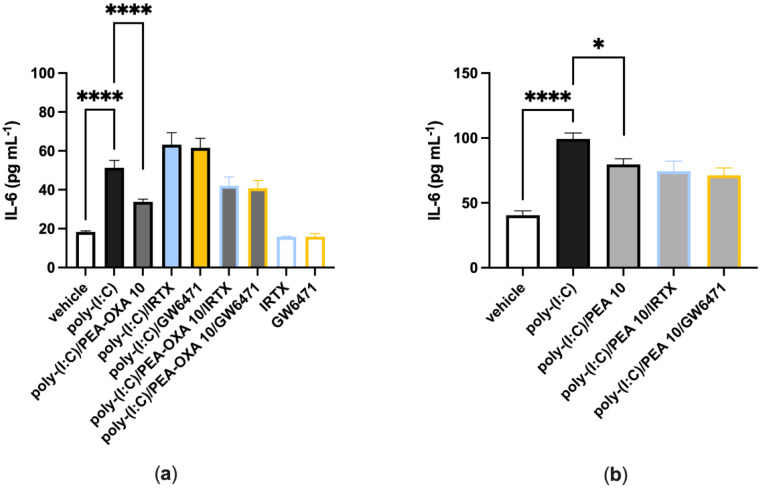
TRPV1 and PPAR-α antagonists do not revert the anti-inflammatory effect of PEA-OXA and PEA in poly-(I:C)-stimulated A549 cells. (**a**) IL-6 release was measured after that A549 cells were stimulated with poly-(I:C) (100 μg mL^−1^) and treated with IRTX (0.1 μM) or GW6471 (1 µM) in the presence or absence of PEA-OXA (10 μM), for 6 h at 37 °C in a 5% CO_2_ atmosphere; (**b**) IL-6 release was measured after A549 cells were stimulated with poly-(I:C) (100 μg mL^−1^) and treated with IRTX (0.1 μM) or GW6471 (1 µM) in the presence or absence of PEA (10 μM), for 6 h at 37 °C in a 5% CO_2_ atmosphere. Each bar shows the mean ± SEM of independent experiments (*n* = 4). The *p*-values were determined by ANOVA followed by Tukey’s multiple comparisons test. **** *p* < 0.0001 and * *p* < 0.05.

When A549 cells were stimulated with poly-(I:C) (100 μg mL^−1^ for 6 h) and treated with a selective TRPV1 (IRTX, 0.1 µM) or PPAR-α (GW6471, 1 µM) receptor antagonist, IL-6 release was comparable to that observed in poly-(I:C)-stimulated A549 cells treated with the vehicle (Figure 3a). When poly-(I:C)-stimulated A549 cells were co-treated with PEA-OXA (10 μM) and IRTX (0.1 μM) or GW6271 (1 µM), IL-6 release was comparable to that observed in poly-(I:C)-stimulated A549 cells treated with PEA-OXA (10 μM) (Figure 3a). Likewise, when poly-(I:C)-stimulated A549 cells were co-treated with PEA (10 μM) and IRTX (0.1 μM) or GW6271 (1 µM), IL-6 release was comparable to that observed in poly-(I:C)-stimulated A549 cells treated PEA (10 μM) (Figure 3b). No effect was observed on IL-6 release when A549 cells were treated with the antagonists alone, i.e., in the absence of poly-(I:C), as compared to vehicle-treated A549 cells (Figure 3a).

### 3.3. Effect of PEA and PEA-OXA Treatment on AEA, 2-AG and PEA Endogenous Levels, in Poly-(I:C)-Stimulated A549 Cells

**Figure 4:**
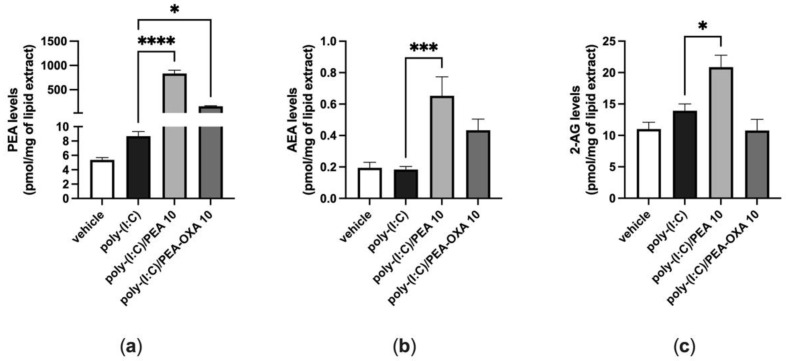
Variation of the levels of PEA, AEA and 2-AG in poly-(I:C)-stimulated A549 cells treated with PEA and PEA-OXA. PEA (**a**), AEA (**b**) and 2-AG (**c**) levels were quantified by LC–MS; after that, A549 cells were stimulated with poly-(I:C) (100 μg mL^−1^) in the presence or absence of PEA (10 μM) and PEA-OXA (10 μM) for 6 h at 37 °C in a 5% CO_2_ atmosphere. Each bar shows the mean ± SEM of independent experiments (*n* = 3). The *p*-values were determined by ANOVA followed by Tukey’s multiple comparisons test. **** *p* < 0.0001, *** *p* < 0.001 and * *p* < 0.05.

When A549 cells were stimulated with poly-(I:C) under the same conditions shown above (100 μg mL^−1^ for 6 h), the endogenous levels of AEA, 2-AG and PEA did not change, as compared to those in A549 cells stimulated with vehicle (Figure 4). By contrast, when poly-(I:C)-stimulated A549 cells were treated with PEA (10 μM), the endogenous levels of AEA and 2-AG were significantly increased by 4-fold and 1.5-fold, respectively, compared to those in poly-(I:C)-stimulated A549 cells treated with the PEA vehicle (Figure 4b,c). In addition, the levels of PEA were significantly increased by 96-fold when poly-(I:C)-stimulated A549 cells were treated with PEA (10 µM), as compared to those in poly-(I:C)-stimulated A549 cells treated with the vehicle of PEA (Figure 4a). The endogenous levels of PEA were significantly increased by 18-fold when poly-(I:C)-stimulated A549 cells were treated with PEA-OXA (10 µM), as compared to those in poly-(I:C)-stimulated A549 cells treated with the vehicle of PEA-OXA (Figure 4a). No statistically significant variation in the endogenous levels of AEA and 2-AG was observed when poly-(I:C)-stimulated A549 cells were treated with PEA-OXA (10 µM) (Figure 4a), compared to poly-(I:C)-stimulated A549 cells treated with the PEA-OXA vehicle, although a trend towards the enhancement of the AEA levels was observed (Figure 4a).

### 3.4. PEA-OXA and PEA Block TGF-β-Induced Epithelial–Mesenchymal Transition in Lung Epithelial Cells

**Figure 5:**
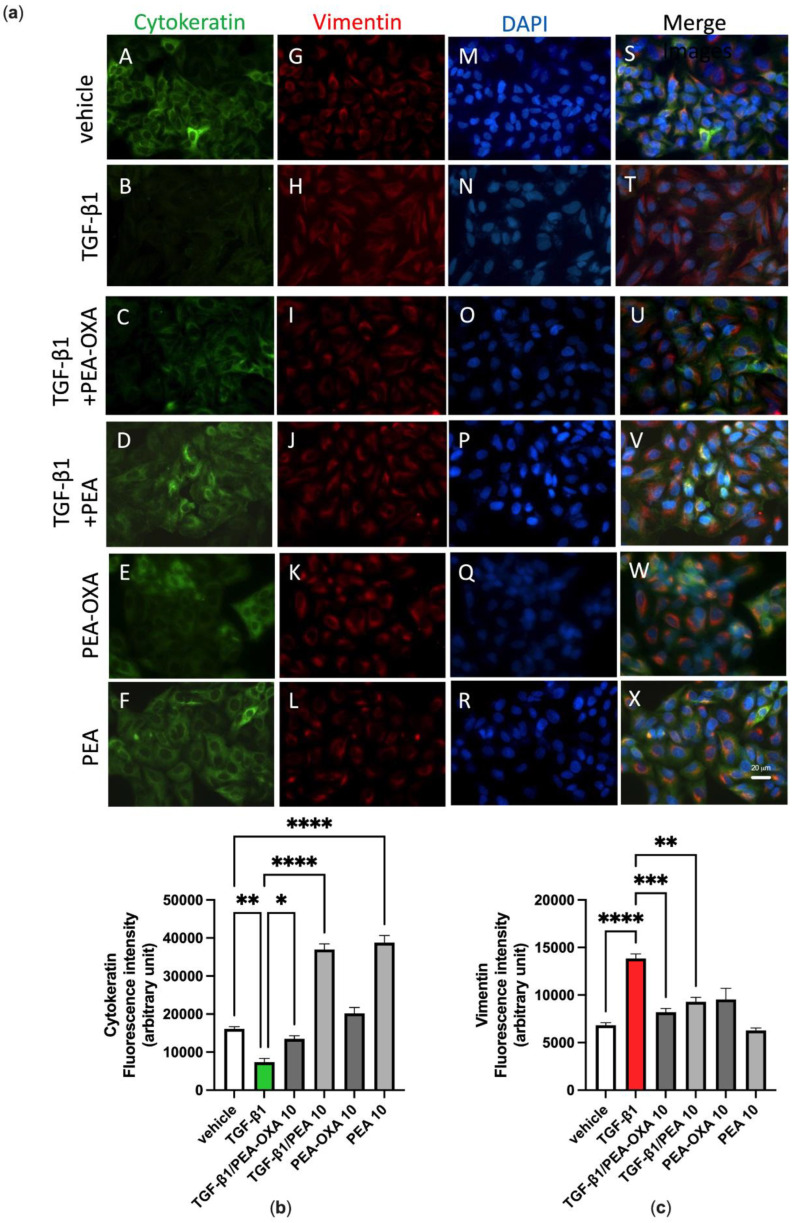
TGF-β1-induced epithelial–mesenchymal transition in A549 cells as evidenced by immunofluorescence approaches is avoided by the treatment with PEA-OXA or PEA. (**a**) Immunofluorescence staining was examined for the following markers: cytokeratin, an epithelial marker (green fluorescence, panels A–F), and vimentin, a mesenchymal marker (red fluorescence, panels G–L). DAPI staining was included to visualize the cell nucleus (blue fluorescence, panels M–R). In the merged images the co-expression and co-distribution of the markers are visualized (panels S–X). Quantifications of cytokeratin (**b**) and vimentin (**c**). The cells were captured with a 40 × microscope objective (Bar = 20 µm). Each bar shows the mean ± SEM of independent experiments (*n* = 3). The *p*-values were determined by ANOVA followed by Tukey’s multiple comparisons test. **** *p* < 0.0001, *** *p* < 0.001, ** *p* < 0.01 and * *p* < 0.05.

We previously reported that A549 cells cultured in the absence of TGF-β1 maintain a classic epithelial morphology appearing short, spindle-shaped and triangle-shaped, whereas following incubation with TGF-β1 (2 ng mL^−1^) for 72 h, these cells assume an elongated shape, with many cells losing contact with their neighboring cells and displaying a long spindle shape typical of a fibroblast-like morphology, an effect known as Epithelial–Mesenchymal Transition (EMT) [32]. Consistent with these morphological observations, alterations in the expression and distribution of cytokeratin and vimentin were evidenced here by immunofluorescence assays. Indeed, these observations showed that, under the same experimental conditions, TGF-β1 induced the down-regulation of cytokeratin (Figure 5a, panels B and T; Figure 5b) and the up-regulation of vimentin (Figure 5a, panels H and T; Figure 5c), when compared to vehicle-incubated A549 cells (Figure 5a, panels A, G and S; Figure 5b,c). Treatment of TGF-β1-incubated A549 cells with PEA-OXA (10 µM) or PEA (10 µM) reverted the down-regulation of cytokeratin (Figure 5a, panels C, U, D and V; Figure 5b) and the up-regulation of vimentin (Figure 5a, panels I, U, J and V; Figure 5c). No cell alteration in the expression and distribution of cytokeratin and vimentin was evidenced by immunofluorescence assay when A549 cells were treated with PEA-OXA (10 µM) alone (Figure 5a, panels E, K and W; Figure 5b,c), i.e., in the absence of TGF-β1, as compared to vehicle-treated A549 cells (Figure 5a, panels A, G and S; Figure 5b,c). Likewise, no cell alteration in the expression and distribution of vimentin was evidenced by immunofluorescence assay when A549 cells were treated with PEA (10 µM) alone (Figure 5a, panels L and X; Figure 5c), as compared to vehicle-treated A549 cells (Figure 5a, panels G and S; Figure 5c). Instead, PEA (10 µM) alone showed a per se effect on the expression and distribution of cytokeratin (Figure 5a, panels F and X; Figure 5b), as compared to vehicle-treated A549 cells (Figure 5a, panels A and S; Figure 5b).

## 4. Discussion

Since the effectiveness of ultra-micronized PEA i) in attenuating acute lung inflammation, reducing immune cell infiltration and cytokine release in an acute lung injury model induced by lipopolysaccharide (LPS) [33] and ii) in inhibiting the pro-inflammatory response activated by SARS-CoV-2 spike protein in cultured murine alveolar macrophages has been already demonstrated [28], in this study we investigated for the first time if PEA-OXA, a congener of PEA endowed with anti-inflammatory activity [23], could be as effective as PEA in counteracting the IL-6 production and lung cell failure typical of COVID-19. For this purpose, we used two different in vitro models of acute lung injury. In the first in vitro model, we used a human lung epithelial cell line (A549) activated by a stable synthetic double-stranded RNA (poly-(I:C)) that can bind to toll-like receptor 3 (TLR3) with high affinity [34] to stimulate the pathophysiological viral disease state and reproduce the cell signaling pathways typical of the cytokine storm in terms of IL-6 overproduction. Our results demonstrated that both PEA-OXA and PEA were able to counteract IL-6 release induced by poly-(I:C) in A549 cells. Interestingly, PEA-OXA resulted to be more efficacious than PEA (at the same concentration tested and in terms of percent of the effect of poly-(I:C) alone) at counteracting poly-(I:C)-induced IL-6 cytokine release. It is possible that PEA-OXA exerts a stronger protective effect against IL-6 than PEA because it might act through a dual mechanism of action, both PEA-mediated and PEA-independent. In fact, we also investigated the mechanism of action through which PEA-OXA and PEA could exert their anti-inflammatory effects in A549 cells. Our results indicated that the inhibitory effect of PEA-OXA and PEA on poly-(I:C)-induced IL-6 cytokine release was not reverted by an antagonism at TRPV1 and PPAR-α receptors, although these receptors, and particularly the latter one, were strongly expressed in A549 cells. This suggests a non- TRPV1- and non-PPAR-α-mediated mechanism for the actions of the two molecules. Therefore, we investigated the ability of PEA-OXA and PEA to modulate the endogenous levels of endocannabinoids (AEA and 2-AG), as well as, in the case of PEA-OXA, the endogenous levels of PEA, in poly-(I:C)-stimulated A549 cells. Our results confirmed the existence of an “entourage” effect of PEA on the endogenous levels of 2-AG [15] and demonstrated for the first time the ability of PEA to also increase the endogenous levels of AEA in an inflammatory condition. In addition, we confirmed the ability of PEA-OXA to increase the endogenous levels of PEA in inflammatory conditions, an effect that might be exerted by inhibiting the enzyme responsible for PEA degradation (i.e. NAAA) [23]. In line with this hypothesis, we found a strong mRNA expression of NAAA in untreated A549 cells. This finding was not surprising, since NAAA is known to be abundantly expressed in lung cells and tissues [35]. Intriguingly, despite the fact that PEA-OXA significantly (18-fold) elevated the endogenous levels of PEA, this was not sufficient for this compound to also trigger the elevation of the endocannabinoid levels, suggesting that the elevation of PEA cellular levels beyond a certain threshold is necessary to induce an “entourage” effect. Indeed, and not surprisingly, exogenous PEA administration to the cells caused a much higher elevation of the cellular PEA levels (96-fold) than PEA-OXA at the same concentration. These results, taken together, suggest that the anti-inflammatory effects of PEA-OXA and PEA may be partially mediated by bioactive endogenous lipids (i.e., endocannabinoids in the case of PEA, and PEA in the case of PEA-OXA). Further studies will be needed to further clarify the mechanism(s) of the anti-inflammatory actions described for PEA and PEA-OXA in lung cells.

In the second in vitro model, we used A549 cells activated by TGF-β in order to reproduce the lung fibrosis that is a critical feature of chronic lung diseases and a serious complication of SARS-CoV-2 infection. In particular, TGF-β modulates lung tissue morphogenesis and differentiation by inducing the development of EMT, an important cellular process in chronic respiratory diseases. Our results suggest that following exposure to TGF-β, A549 cells acquire a fibroblast-like morphology characterized by a decrease of the epithelial marker expression and an increase of the mesenchymal marker expression. The immunofluorescence analysis showed the presence of EMT, characterized by a reduction in cytokeratin-positive staining and an increase in vimentin-positive staining, which was inhibited by the treatment with both PEA-OXA and PEA. These results are in agreement with previous data, in which ultra-micronized PEA inhibited the inflammation response and lung fibrosis in mice subjected to idiopathic pulmonary fibrosis [36].

## 5. Conclusions

In summary, in this study we reported for the first time the protective effects of PEA-OXA and PEA in counteracting the inflammatory response induced by poly-(I:C), as well as in reverting the fibrosis induced by TGF-β. Our results also evidence a greater effectiveness of PEA-OXA over PEA and point to PEA-OXA as a new and promising candidate in the management of acute lung injury caused by conditions induced by a cytokine storm. 

## Figures and Tables

**Table 1:** List of primer sequences used in the qPCR analysis.

*gene*	Forward (5′–3′)	Reverse (5′–3′)
**CNR2**	TAGTGCTGAGAGGACCCA	CGCTATCCACCTTCCTACAA
**TRPV1**	CTGCCCGACCATCACAGTC	CTGCGATCATAGAGCCTGAGG
**PPAR** **α**	TTCGCAATCCATCGGCGAG	CCACAGGATAAGTCACCGAGG
**NAAA**	TGACAGTGGATGTGCAATTCTT	GCCTTTATCTCGTTCATCACCAG
**S16**	TCGGACGCAAGAAGACAGCGA	AGCGTGCGCGGCTCAATCAT

## Data Availability

Not applicable here.
